# Perinatal testicular torsion diagnosed as an intraperitoneal mass: case report and literature review

**DOI:** 10.3389/fped.2025.1501645

**Published:** 2025-02-26

**Authors:** Cheng Huang, Yan Ma, Dongsheng Bai, Chunsheng Hao

**Affiliations:** Department of Urology, Children’s Hospital, Capital Institute of Pediatrics, Beijing, China

**Keywords:** perinatal testicular torsion, cryptorchidism, intraperitoneal mass, case report, diagnose and treatment

## Abstract

Perinatal testicular torsion is not common in clinic. We report our experience in treating such a condition in a 50-day boy with an intraperitoneal mass. The clinical manifestations, physical examination, imaging examination and treatment of the boy were retrospectively analyzed and the related literatures were reviewed. Laparoscopic exploration, resection of abdominal mass, high ligation of left processus vaginalis and left orchiopexy were performed after admission. According to the results of intraoperative frozen-section examination and paraffin section after operation, combined with the history of children, consider the diagnosis of “right cryptorchidism with perinatal testicular torsion”. We reported the case for the purpose of exploring the characteristics, diagnosis, differential diagnosis and treatment of perinatal testicular torsion (Perinatal testicular torsion, PTT), and improving the understanding of cryptorchidism with perinatal testicular torsion.

## Introduction

Testicular torsion (Testicular Torsion, TT) is a common emergency in pediatric urology, and its incidence has obvious “bimodal distribution” in newborn and adolescent children (1). However, perinatal testicular torsion (PTT) is relatively rare, with an incidence rate of about 6.1/10,000 (2). PTT is generally roughly defined as testicular torsion that occurs before birth or within 30 days after birth (3), which is called prenatal testicular torsion and postpartum testicular torsion, respectively. PTT accounts for about 10% of testicular torsion children (4). PTT is mainly extravaginal torsion. Macrosomia, breech delivery, prolonged labor and dystocia are considered to be risk factors for PTT (5). The pathogenesis, treatment, and surgical timing of perinatal testicular torsion are currently controversial.

This study reports a case of perinatal testicular torsion diagnosed as an intraperitoneal mass treated in our center. The clinical manifestations and signs of PTT are often atypical, so it is sometimes difficult to make a timely and accurate diagnosis. When perinatal testicular torsion combined with cryptorchidism, clinical diagnosis is more difficult and prone to misdiagnosis. When scrotal emptiness along with an ipsilateral intraperitoneal mass occurs, we hope to highlight the importance of considering the diagnosis of perinatal testicular torsion, so as to make a timely and accurate diagnosis and avoid the delay of treatment.

## Case presentation

**Figure 1:**
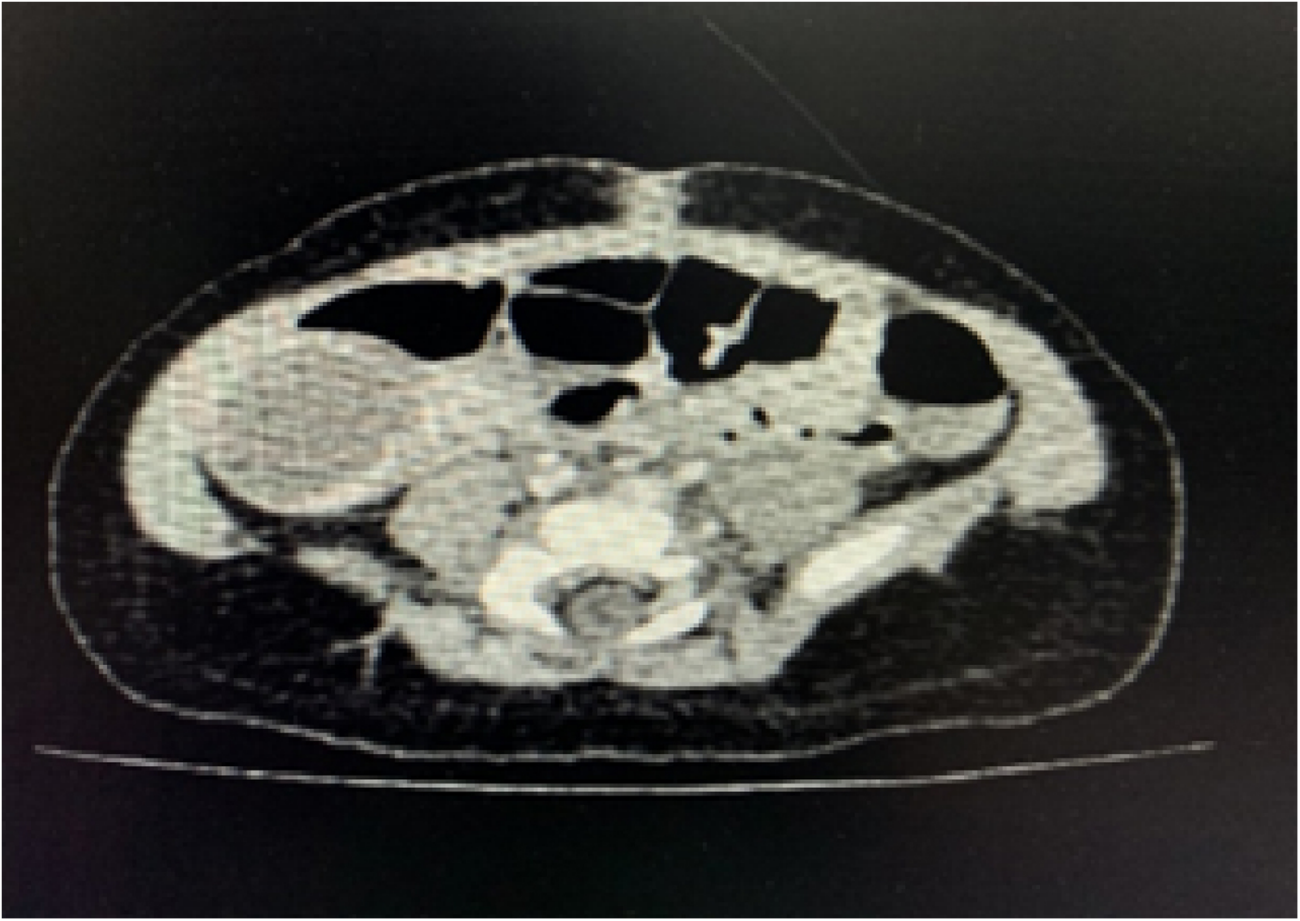
Abdominal enhancement CT: oval-like cystic mass was seen in the right lower quadrant, with a size of 25*30*22 mm. The CT value of the mass was about 30 HU, with a strip of high density shadow at the edge.

The child, male, 50 days, with full-term natural delivery, was found to have a cystic mass in the right abdomen by ultrasound during routine prenatal examination at 37 weeks of gestation, and it was suggested to follow up with ultrasound at 1 month after birth. The child was generally in good condition after birth and had been admitted to our hospital for further treatment. Physical examination: External genital of normal male infants; Normal penile development; Right scrotum was empty and poorly developed; The right testis was not palpable in the scrotum; There was no tender and no testicular-like tissue in the right inguinal region; The left testis was located within the scrotum and well developed. The abdomen was soft and no significant mass was palpable. Routine prenatal examination and B-ultrasound at 37 weeks of pregnancy revealed a cystic mass in the right abdominal cavity of the fetus. B-ultrasound of reproductive system (48 days after birth): No clear testicular echo was detected in the right scrotum. No right testicle-like echo was detected in the right groin, perineum, symphysis pubis and in the pelvic cavity. The left testis was 1.34*0.64*0.83 cm in size and located within the scrotum with no obvious morphological abnormalities. A very small amount of effusion was seen above the left testis, about 0.34 cm in thickness. A mass was visible on the right lower quadrant which was quasi-circular, about 2.9*2.8*3.6 cm in size. The internal echo was significantly uneven, which was a small shadow of medium and strong echo mixed with low echo. No obvious fluid or calcification was found inside the mass. The capsule of the lesion was thick, about 0.28 cm in thickness. CDFI: Visible blood flow signal in the capsule and no obvious blood flow signal inside. Abdominal contrast-enhanced CT (Figure 1): Oval-like cystic mass was seen in the right lower quadrant, with a size of 25*30*22 mm. The CT value of the mass was about 30 HU, with a strip of high density shadow at the edge. The right scrotum is empty with no visible testicular structure. Testicular structure was seen in the left scrotum and the enhancement was not even. Tumor markers: HCG: <0.1 mIU/ml (normal range: ≤2.0 mIU/ml), CEA: 2.41 ng/ml (normal range: ≤5.0 ng/ml), AFP: 1,212.21 ng/ml (normal range: 25–999.99 ng/ml).

**Figure 2:**
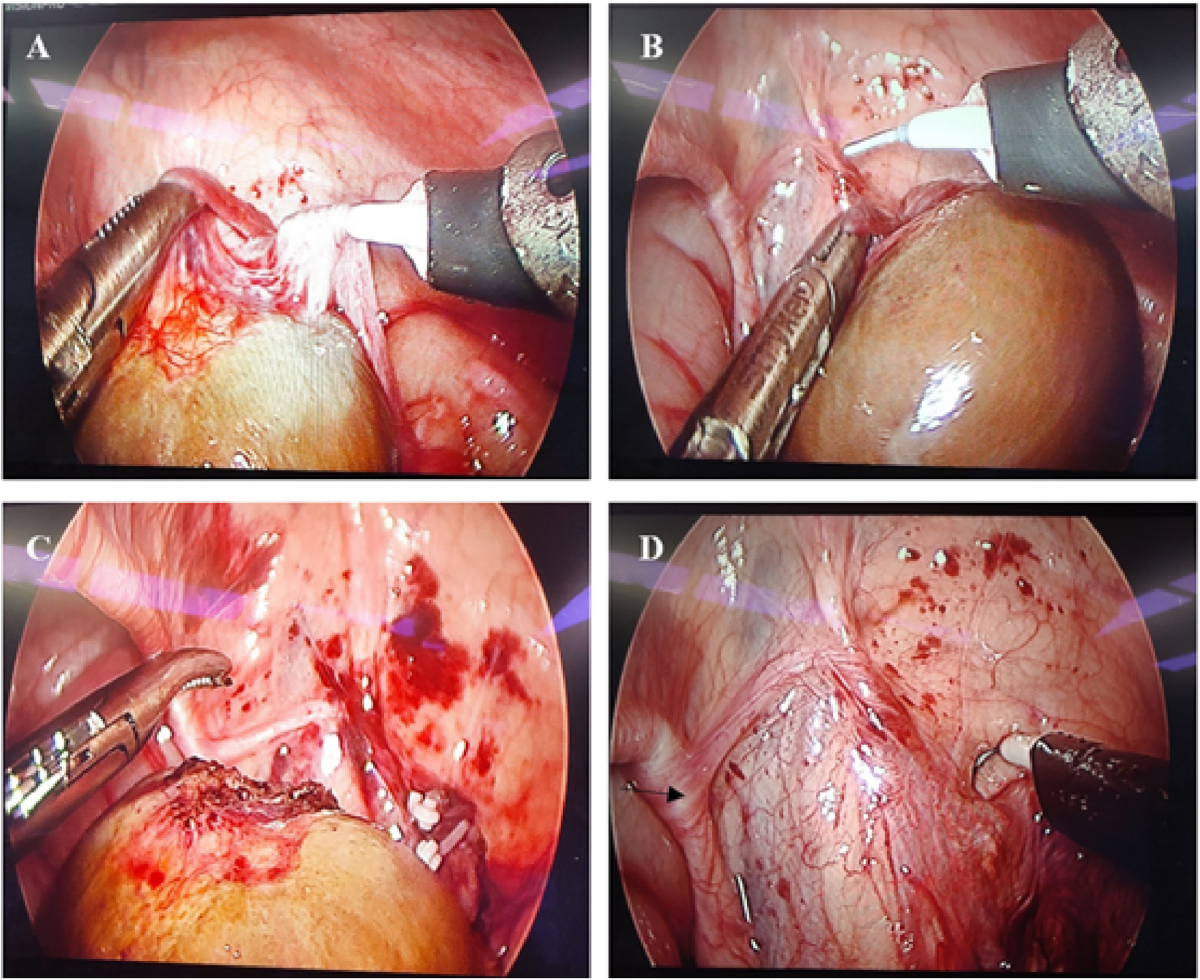
**(A)** during the operation, there was a mass in the right lower quadrant, and the mass was dark brown and about 3 cm in diameter. **(B)** Dissociate the blood vessels supplying the mass (Torsion was seen in the spermatic cord vessels. The torsional part was slender with severe adhesion. The degree of torsion couldn't be assessed.) **(C)** Sever the blood vessels supplying the mass. **(D)** Local appearance of the right lower right lower quadrant after complete resection of the mass (The arrow points to the vas deferens).

**Figure 3:**
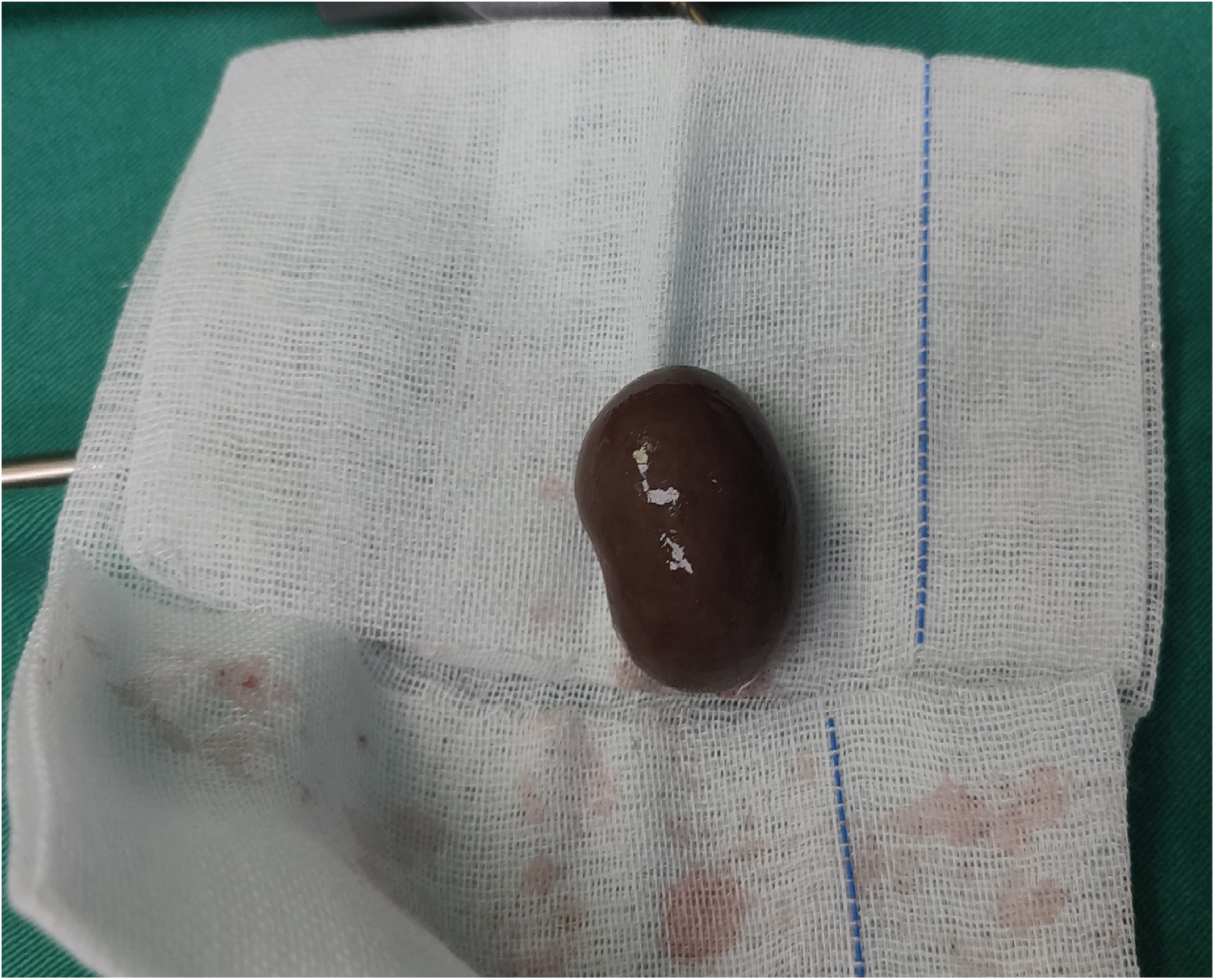
The appearance of the mass in the right lower abdomen after complete resection, unilateral depression like broad bean shape, dark brown.

A series of routine examinations were performed before surgery, and the results showed that the child could tolerate surgery under general anesthesia. Laparoscopic exploration was performed under general anesthesia. We took a longitudinal incision of umbilical 1.5 cm in length. Three laparoscopic trocars were implanted. A 10 mmHg pneumoperitoneum was established. We explored and found a mass in the right lower quadrant, which was about 3 cm in diameter, dark brown (Figures 2, 3). The left processus vaginalis was found to be unclosed, with a diameter of about 0.8 cm. We fully dissociated the three blood vessels supplying the mass(Spermatic vessels, gubernaculum vessels and vas deferens vessels). Torsion was seen in the spermatic cord vessels. The torsional part was slender with severe adhesion. The degree of torsion couldn't be assessed. We used hem-o-lock to clamp off the vessels and then severed it. We sent the retrieval bag through the umbilical Trocar and put the mass in. The umbilical incision was extended vertically, and the mass was removed through the umbilical incision. The mass was sent to the frozen section for pathology. Pathological report showed extensive bleeding, degeneration and necrosis of tissue, with diffuse acute and chronic inflammatory cell infiltration and focal calcification, and no active and tumor components were found in frozen tissue. Combined with the history of the child, it was possible to consider cryptorchidism with perinatal testicular torsion. Laparoscopic high ligation of the left unclosed processus vaginalis and left orchiopexy was performed and ended the operation. The results of postoperative paraffin section suggested that there were necrotic tissue with extensive hemorrhage and diffuse acute and chronic inflammatory cell infiltration, and focal scattered calcification. No active components and tumor lesions were found.

Up to now, the child has been regularly reexamined two and a half years after surgery. The latest telephone follow-up results showed that the child's height and weight development were normal, and the contralateral testis was located in the scrotum and well-developed.

## Discussion

At present, there is a great controversy about the treatment of PTT, mainly focusing on the emergency operation or delayed surgical exploration, whether to retain the twisted testis and whether the contralateral uninvolved testicles need preventive fixation. The diagnosis of PTT combined with intraperitoneal cryptorchidism remains challenging due to the rarity of cases. In this study,we report a boy with PTT combined with intraperitoneal cryptorchidism diagnosed as an intraperitoneal mass at first.We hope to improve the understanding of the condition and avoid the delay of treatment.

The etiology of perinatal testicular torsion is not clear, and some think that it is related to two factors: one is the loose connection between the tunica vaginalis and the dartos fascia; The second is the overactive cremasteric reflex (6). For the latter, it is generally believed that both the third trimester of pregnancy and the process of vaginal birth can lead to an increase in uterine pressure, which can stimulate the contraction of the cremaster and induce testicular torsion, such as macrosomia, breech birth, prolonged labor and dystocia. It has also been suggested that complex pregnancies can lead to congenital testicular hypoplasia in newborns, thus increasing the risk of testicular torsion in newborns (7).

Testis have poor tolerance to ischemia and hypoxia. Generally speaking, testicular ischemia can cause irreversible damage within 4–8 h (8). Therefore, early detection and early treatment are very important for the rescue of ischemic testis. According to the time of torsion, perinatal testicular torsion can be divided into prenatal testicular torsion and postpartum testicular torsion, of which prenatal testicular torsion accounts for about 70%–80% (9). Clinical manifestations are usually related to the duration of testicular torsion. According to clinical manifestations, perinatal testicular torsion is usually divided into emergency type and non-emergency type (10). The emergency type is characterized by enlarged scrotum, dark red or purplish black scrotum, usually accompanied by tenderness, sometimes accompanied by restlessness or refusal to feed. During physical examination, the disappearance of cremasteric reflex in this age group is sometimes unreliable (5). The non-emergency type showed that the appearance of the local skin of the scrotum was basically normal, the abnormal mass of the scrotum could be touched, there was no tenderness, and sometimes the affected side of the testicle can also be found absent. Due to the variety of clinical manifestations of perinatal testicular torsion, which is usually related to the duration of testicular torsion and lack of specificity, sometimes hydrocele or edema in the neonatal period will cover up the clinical manifestations of perinatal testicular torsion, relying solely on clinical manifestations and physical examination, it is easy to cause missed diagnosis or misdiagnosis. In this study, the children only showed the emptiness of the scrotum on the affected side and the cystic mass of the right abdominal cavity found by ultrasound during pregnancy, but there were no other specific manifestations. Currently, ultrasonography has gradually become an important diagnostic tool for perinatal testicular torsion.

Ultrasonography can timely detect the blood flow in the testis and the morphology of the testis, which plays an important role in the early diagnosis of testicular torsion. For perinatal testicular torsion, the morphology and blood perfusion of testis on B-ultrasound are closely related to the duration of testicular torsion (4). Normally, testicular tissue is homogeneous echo with blood flow signals inside. In the acute stage of perinatal testicular torsion, B ultrasound can show “double ring bleeding”, that is, the enlarged testis and epididymis can be surrounded by two bloody fluid distributed in concentric circles, and the thickened and edema tunica albuginea can be observed, sometimes with subalbuginea effusion or hydrocele (4). It has been suggested that hydrocele may be an early sign of testicular torsion, which may be related to vascular changes or local inflammatory response (11). With the increase of testicular ischemia duration, testicular atrophy can be observed in B ultrasound. Sometimes, hyperechoic ring structure will appear in the periphery of the testis, which is the calcification between the testicular parenchyma and the transition area of the tunica albuginea. Usually, the non-acute signs of torsion calcification will be more obvious. Generally, the presence of calcification usually suggests that testicular torsion has been present for at least several weeks (4). Some believe that there is a significant difference in ultrasonic manifestations between acute and non-acute PTT, especially in testicular volume and echo homogeneity. The proportion of blood flow signal disappearance in non-acute PTT is high, accounting for 94.1% and 64.3% in acute respectively (12). In this case, B-ultrasound of reproductive system showed that no clear testicular echo was detected in the right scrotum and a mass was visible on the right lower quadrant which was quasi-circular. The internal echo of the mass was significantly uneven, which was a small shadow of medium and strong echo mixed with low echo. No obvious fluid or calcification was found inside the mass. CDFI: Visible blood flow signal in the capsule and no obvious blood flow signal inside. The ultrasonographic manifestation of this case is similar to that of non-emergency PTT. Although ultrasound is an important tool for perinatal testicular torsion, it can be distinguished from epididymitis, hydrocele, indirect inguinal hernia, testicular mass, meconium peritonitis and other diseases (4). However, it can only be used as an important auxiliary examination and can not replace surgical exploration.

Currently, the main treatment methods for perinatal testicular torsion are surgery and conservative treatment. There is controversy about the timing and urgency of surgical treatment, whether the affected side of the testis needs to be removed and whether the contralateral testis needs to be fixed. Some studies have shown that prenatal PTT accounts for about 70%–80% (9). Melcer et al. (6) believe that most of the prenatal PTT can only be diagnosed after delivery, the duration of testicular torsion is longer, and the possibility of testicular salvage is very small, but the contralateral uninvolved testis must be closely observed and followed up to avoid asynchronous torsion. On the contrary, Monteilh et al. (13) believe that emergency exploration of bilateral testis and fixation of the contralateral uninvolved testis should be recommended to avoid anorchidism. Similarly, Wang et al. (14) believe that although the testicular retention rate of emergency surgery is low, there is still the possibility of testicular survival, and they suggest active surgical exploration and testicular fixation. The results of the study by Niu et al. (10) showed that there was no normal testicular tissue structure in the scrotum of the affected side of PTT, which was a hard mass, adhesion with the surrounding tissue, and the direction and degree of torsion were not clear. The postoperative pathological results showed that most of the hemorrhage, necrosis and calcification, and the testicular structure had been basically lost. However, the pathological examination of postpartum PTT showed that there were residual seminiferous tubules and vas deferens, suggesting that emergency surgical intervention may be of certain significance to the preservation of testicular function. In the study of Abraham et al. (9), there were 3 children with perinatal bilateral testicular torsion who underwent surgical exploration within 8 h after admission, underwent orchiectomy on the side with severe testicular torsion, and orchiopexy was performed after the contralateral testicular detorsion. One case of testicular atrophy and disappeared during the follow-up, and the testicular size of the other two cases returned to normal at 1 year old and 21 months respectively. Kaye et al. (15) believe that prenatal PTT can lead to irreversible testicular damage, which is almost irreparable, and the salvage rate is usually only 5%, so there is no need for emergency intervention, while postpartum PTT can sometimes be saved and requires active surgical treatment. Melcer et al. (6) retrospectively analyzed 36 cases of unilateral prenatal testicular torsion. All the testis were found to be necrotic during exploration and the orchiectomy was performed. In their study, Niu et al. (10) proposed the main view of PTT treatment. For testicular torsion clearly occurring postpartum, emergency surgery should be actively performed to save the testis in the affected side as much as possible. For children aged 6–8 weeks, the gubernaculum and tunica vaginalis are in the process of fixation with the scrotum, testicular torsion is easy to occur, and the contralateral testis is at a high risk of possible torsion, so the unilateral PTT found at this time, whether prenatal or postpartum, should be operated actively and the contralateral testis should be fixed. After 8 weeks of age, the possibility of further testicular torsion is significantly reduced, and most of the PTT are prenatal type, which is difficult to rescue even after emergency surgery. Therefore, children at this stage have been identified as prenatal PTT, considering the necrotic testis, no emergency surgical exploration is necessary, and active clinical observation of the contralateral testis is needed. For the ischemic and necrotic testis in the exploration of unilateral PTT, orchiectomy is recommended. On the one hand, the necrotic testis may cause infection or organization, on the other hand, because the integrity of the blood-testicular barrier is disrupted, the body produces antisperm antibodies that affect sperm quality and may affect contralateral testis function (16). However, Cuervo et al. (17) thought that the immature spermatogenic tissue in the neonatal period could not provide enough antigen stimulation, so they could also choose to retain the ischemic and inactive testis *in situ*, and Callewaert et al. (3) suggested that the testes could be preserved even if the perfusion was not significantly restored after testicular detorsion, because there may be some living tissue in the twisted testis, and Leydig cells seem to be more resistant to ischemia and hypoxia. Therefore, some endocrine functions may be retained. As for the surgical approach, most scholars use scrotal incision exploration, but some scholars still use inguinal exploration because there is less possibility of finding tumors during the operation. However, this surgical approach may increase the potential possibility of spermatic cord and vascular injury and postoperative hydrocele and indirect inguinal hernia (5). Some studies also believe that if inguinal exploration is used on the affected side, then the contralateral side should be explored through the scrotum, and the testicles should be fixed in the Dartos fossa between the external spermatic fascia and Dartos fascia, which is less traumatic (8).

Due to the popularity of ultrasound technology, the detection rate of prenatal PTT is gradually increasing. For those who consider prenatal PTT during pregnancy, some scholars (6) suggest the following: (1) For unilateral testicular torsion, if ultrasound has evidence of an acute torsion within 24 h and the gestational age exceeds 34 weeks, immediate induction of labor and postnatal surgical intervention can be performed, or follow-up can be continued, follow obstetrical instructions for delivery. (2) For bilateral testicular torsion, if the gestational age is less than 34 weeks, follow the obstetric indications. If the gestational age is more than 34 weeks, immediate induction of labor and postnatal surgical intervention can be performed.

Perinatal testicular torsion is relatively rare and clinical manifestations and signs are often atypical, so it is sometimes difficult to make a timely and accurate diagnosis. In the case presented in this study, according to the patient's medical history, the fetus had a cystic mass in the right abdomen during pregnancy, and the physical examination results were consistent with the manifestation of right cryptorchidism. Combined with the results of abdominal ultrasound, the preliminary diagnosis of “right cryptorchidism and right intraabdominal mass” was considered. After admission, laparoscopic exploration, resection of abdominal mass, high ligation of left processus vaginalis and left orchiopexy were performed. The patient recovered well and the left testis was well-developed. Pathological sections showed no active components and tumor lesions. The pathological findings are highly consistent with the pathological findings of non-acute perinatal testicular torsion reported by Niu et al. (10). A review of the preoperative abdominal B-ultrasound results showed that there was a round mass in the right lower right lower quadrant, a significantly uneven low echo mixed with medium and strong echo, and the capsule thickness was about 0.28 cm. The results were highly consistent with the sonogram of perinatal testicular torsion reported by Zhou et al. (18) and Tang et al. (19).

The advantage of this study is that the disease is relatively rare, which can provide some guidance for the future diagnosis and treatment. However, this study has limitations. It is under-representative as a case report, and it is unclear to what extent the findings can be generalized to other children with this condition, so we should be mindful of this condition for maximum benefit for children.

## Conclusion

We reported a rare case of PTT combined with intraperitoneal cryptorchidism with an intraperitoneal mass as the initial symptom. PTT with intraperitoneal cryptorchidism should be considered in the differential diagnosis in children who present with an unpalpable testis and an intraperitoneal mass. B ultrasound is the first choice of early diagnosis of PTT. For children considered for prenatal perinatal testicular torsion, although the testicular salvage rate is low, intraoperative exploration and prophylactic fixation of the healthy testis are very important. At present, the prevention, early diagnosis and early treatment of perinatal testicular torsion need to be further studied.

## Data Availability

The original contributions presented in the study are included in the article/Supplementary Material, further inquiries can be directed to the corresponding author.
